# Low-Frequency Electrical Stimulation Optimizes Neurotrophic and Neuroimmune Signaling in Bisvinyl Sulfonemethyl-Based Nerve Guidance Conduits

**DOI:** 10.3390/ijms27093820

**Published:** 2026-04-25

**Authors:** Ching-Feng Su, Chung-Chia Chen, Wei-Cheng Hsu, Ming-Hsuan Lu, Joanna Pi-Jung Lee, Yung-Hsiang Chen, Yueh-Sheng Chen

**Affiliations:** 1 Graduate Institute of Acupuncture Science, Graduate Institute of Integrated Medicine, School of Chinese Medicine, Department of Biomedical Engineering, China Medical University, Taichung 404, Taiwan; luckyboy1002@yahoo.com.tw (C.-F.S.); chken72000@yahoo.com.tw (W.-C.H.); u100030065@cmu.edu.tw (M.-H.L.); 2 Division of Family Medicine, Pingtung Christian Hospital, Pingtung 900, Taiwan; 3 Linsen Chinese Medicine and Kunming Branch, Taipei City Hospital, Taipei 103, Taiwan; dal06@tpech.gov.tw; 4 School of Chinese Medicine for Post Baccalaureate, I-Shou University, Kaohsiung City 840, Taiwan; 5 Division of Plastic Surgery, Department of Surgery, An-Nan Hospital, China Medical University, Tainan 709, Taiwan; rabbitjoanna@gmail.com; 6 Department of Psychology, College of Medical and Health Science, Department of Bioinformatics and Medical Engineering, Asia University, Taichung 413, Taiwan

**Keywords:** peripheral nerve regeneration, electrical stimulation, bisvinyl sulfonemethyl, nerve guidance conduit, gelatin, sciatic nerve, biomaterials

## Abstract

Peripheral nerve injuries involving critical-sized gaps remain a major clinical challenge. Although autologous nerve grafting is considered the gold standard for peripheral nerve repair, its clinical application is limited by the availability of donor nerve tissue and the risk of donor-site morbidity, including sensory deficits and functional impairment. Therefore, nerve guidance conduits (NGCs) have emerged as a promising alternative when combined with bioactive modulation strategies. In this study, we evaluated bisvinyl sulfonemethyl (BVSM)-crosslinked gelatin conduits integrated with electrical stimulation (ES) at different frequencies (0, 2, 20, and 200 Hz) in a rat sciatic nerve defect model over a 4-week recovery period (*n* = 10 per group). Structural regeneration was assessed by morphometric analysis, electrophysiology, macrophage infiltration, CGRP immunoreactivity, retrograde Fluorogold tracing, quantitative PCR of growth factors and inflammatory cytokines, and behavioral testing. Among all stimulation paradigms, low-frequency ES at 2 Hz produced the most pronounced regenerative effects. The 2 Hz group demonstrated significantly greater axon number, axonal density, and regenerated nerve area compared with control and high-frequency groups (*p* < 0.05). Electrophysiological assessments revealed improved nerve conduction velocity, higher MAP amplitudes, and shorter latencies. Enhanced macrophage recruitment and elevated CGRP expression were observed, suggesting coordinated neuroimmune and neurochemical activation. Gene expression analysis indicated upregulation of neurotrophic factors and balanced inflammatory cytokine responses under low-frequency stimulation. In contrast, high-frequency stimulation (200 Hz) failed to enhance overall regeneration and showed reduced axonal metrics, suggesting possible overstimulation-associated suppression. Collectively, these findings demonstrate that BVSM-crosslinked conduits provide a stable and biocompatible regenerative scaffold, and that appropriately tuned low-frequency electrical stimulation (2 Hz) optimally enhances structural, molecular, and functional recovery. The integration of material engineering with bioelectrical modulation represents a promising strategy for next-generation bioelectronic interfaces in peripheral nerve repair.

## 1. Introduction

Peripheral nerve injuries often result in chronic functional deficits, particularly when the nerve gap exceeds 10 mm. Although autologous nerve grafting remains the clinical gold standard for bridging peripheral nerve defects, its clinical application is constrained by several limitations, including limited availability of donor nerves, the need for additional surgical procedures, and the risk of donor-site morbidity such as sensory loss, neuroma formation, and functional impairment. These limitations have driven increasing interest in the development of artificial nerve guidance conduits (NGCs) as alternative strategies for peripheral nerve repair, particularly for critical-sized nerve defects [1,2,3]. A critical requirement for such scaffolds is a biocompatible crosslinking system that ensures mechanical stability while preserving a microenvironment conducive to neuronal survival and signaling. Traditional crosslinkers, such as glutaraldehyde or carbodiimide, are associated with cytotoxicity or may alter the bioactivity of incorporated growth factors [4,5,6,7]. In contrast, bisvinyl sulfonemethyl (BVSM) is a bifunctional crosslinking agent containing reactive vinyl sulfone groups that selectively react with nucleophilic residues, such as primary amines and thiol groups, in biomacromolecules. This Michael-type addition reaction proceeds under mild aqueous conditions, enabling the formation of stable thioether linkages without generating toxic byproducts. Compared with traditional crosslinkers, such as glutaraldehyde or carbodiimide-based systems, BVSM offers improved biocompatibility by avoiding residual aldehyde toxicity and preserving the bioactivity of incorporated growth factors and extracellular matrix components. In addition, BVSM-crosslinked gelatin networks exhibit tunable mechanical strength, enhanced resistance to hydrolytic degradation, and favorable elasticity that more closely mimics native peripheral nerve tissue. These characteristics make BVSM particularly suitable for fabricating nerve guidance conduits that require both structural stability and a permissive microenvironment for axonal regeneration [8,9]. These properties enable the fabrication of NGCs that more closely mimic the viscoelastic characteristics of native peripheral nerve tissue.

However, structural guidance alone is insufficient to achieve optimal functional recovery. Biochemical and electrical cues are also critical determinants of successful nerve regeneration. Electrical stimulation (ES) has been shown to enhance neurite outgrowth, promote Schwann cell migration, and accelerate remyelination [10,11,12]. Therefore, integrating ES into BVSM-based conduits may bridge the gap between structural support and bioelectrical signaling, potentially improving regenerative outcomes.

Nevertheless, the application of electrical stimulation through biodegradable polymer composites introduces additional complexity. Electrochemical reactions at the material–tissue interface may alter polymer stability, accelerate degradation, or modify surface chemistry [13]. Thus, while combining mechanical support with electrical responsiveness presents significant therapeutic opportunities, it also poses challenges for maintaining long-term conduit performance and structural integrity.

We hypothesize that low-frequency electrical stimulation (particularly 2 Hz), when combined with BVSM-crosslinked gelatin nerve guidance conduits, creates an optimized neuroregenerative microenvironment by enhancing neurotrophic signaling and modulating neuroimmune responses, thereby promoting superior structural and functional peripheral nerve regeneration compared with unstimulated or high-frequency stimulation conditions.

In the present study, we systematically evaluated whether BVSM-crosslinked gelatin tubular conduits, particularly when combined with electrical stimulation at different frequencies, could enhance structural and functional peripheral nerve regeneration in a rat sciatic nerve gap model over a 4-week recovery period. Regenerative outcomes were assessed through histomorphometric analysis, macrophage infiltration, retrograde Fluoro-Gold (FG) labeling of dorsal root ganglia (DRG) neurons, electrophysiological measurements, behavioral testing, and histological examination of regenerated tissues.

## 2. Results

### 2.1. Morphological and Surface Characteristics of the BVSM Nerve Conduit

**Figure 1:**
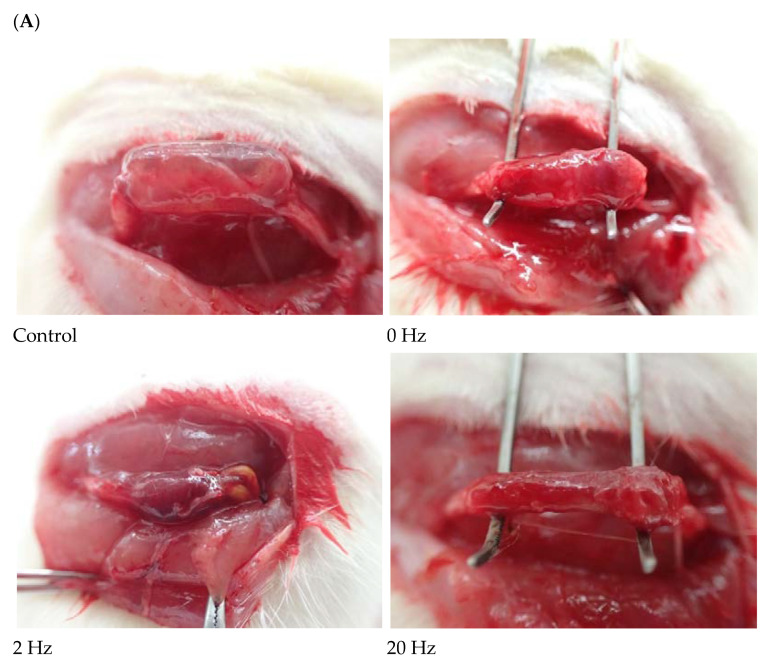

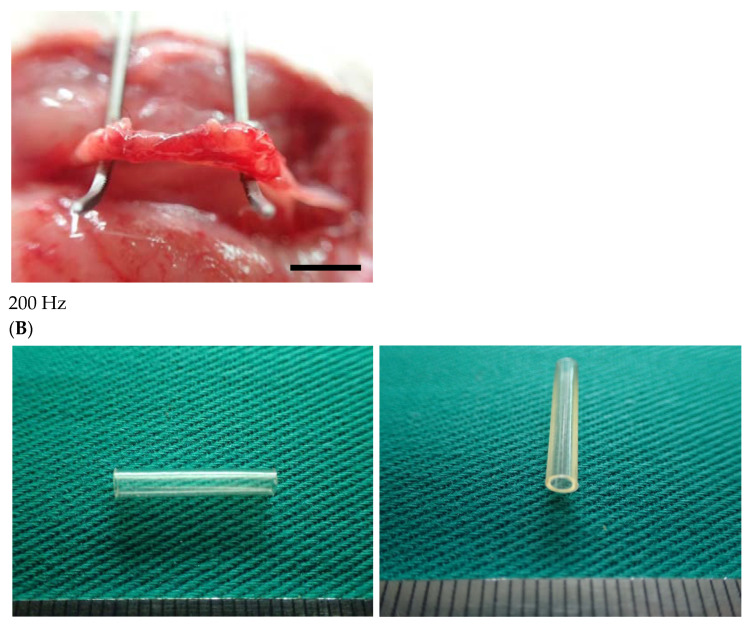
Representative intraoperative images of sciatic nerve repair using BVSM nerve conduits under different ES frequencies. (**A**) Control group without electrical stimulation showing the transected sciatic nerve bridged with the BVSM conduit. BVSM conduit implantation with 0 Hz, 2 Hz, 20 Hz, and 200 Hz ES. In all groups, the proximal and distal nerve stumps were inserted into the lumen of the cylindrical BVSM conduit and secured to maintain alignment across the nerve defect. The conduit maintained structural integrity and appropriate positioning during implantation, demonstrating adequate mechanical stability for bridging the sciatic nerve gap. The black rectangle indicates the scale bar, scale bar = 5 mm. (**B**) Representative images of the BVSM-crosslinked gelatin nerve guidance conduit prior to implantation. Longitudinal view showing the overall morphology of the conduit and cross-sectional view illustrating the hollow lumen structure.

Figure 1 illustrates the BVSM nerve conduit used to bridge transected rat sciatic nerve stumps under electrical stimulation at different frequencies. All conduits exhibited a cylindrical configuration and were positioned between the proximal and distal nerve ends. The nerve stumps were aligned within the conduit lumen, appearing straight and continuous.

The conduit structure remained intact, and the lumen was visibly patent without signs of collapse. Both proximal and distal nerve stumps were inserted into the conduit lumen.

Macroscopically, the outer surface of the BVSM conduit appeared moist, smooth, and glossy, with a semi-transparent appearance. No visible signs of degradation, fragmentation, or discoloration were observed. In addition, no obvious fibrous encapsulation, necrosis, or hemorrhage was observed at the tissue–material interface.

### 2.2. Electrophysiological Recovery Assessed by Nerve Conduction and Muscle Reinnervation

**Figure 2:**
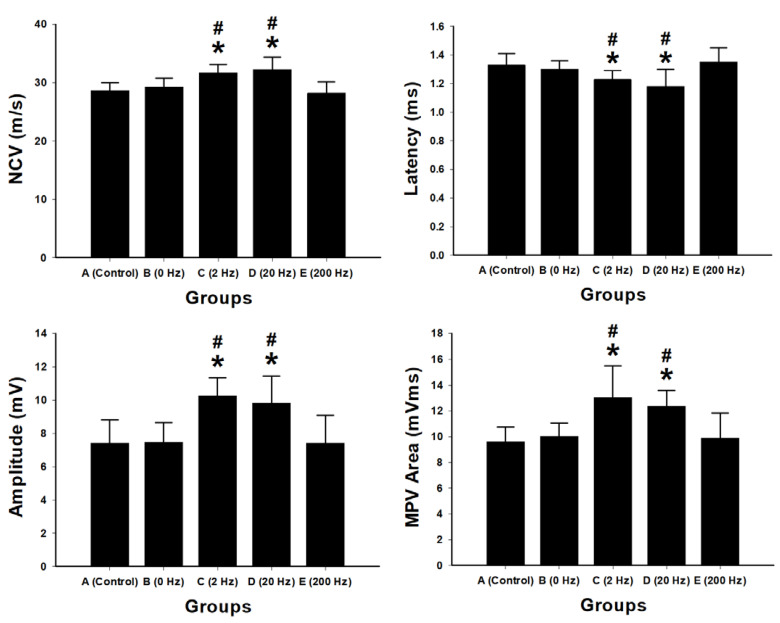
Electrophysiological evaluation of sciatic nerve regeneration following BVSM nerve conduit repair under different ES frequencies. Bar graphs show quantitative analysis of nerve conduction velocity (NCV), latency, compound muscle action potential amplitude, and motor action potential (MAP) area in the control, 0 Hz, 2 Hz, 20 Hz, and 200 Hz stimulation groups. Data are presented as mean ± SD (*n* = 10 per group). Low-frequency ES at 2 Hz and 20 Hz significantly improved electrophysiological parameters compared with the control and 0 Hz groups, demonstrating enhanced nerve conduction and muscle reinnervation. In contrast, high-frequency stimulation at 200 Hz did not produce significant improvements. * *p* < 0.05 vs. control group A; # *p* < 0.05 vs. 0 Hz group B.

Electrophysiological assessments were performed to evaluate nerve conduction and muscle reinnervation following sciatic nerve repair using BVSM nerve conduits under different electrical stimulation frequencies. Measurements included nerve conduction velocity (NCV), latency, compound muscle action potential (CMAP) amplitude, and motor action potential (MAP) area (Figure 2).

The 2 Hz stimulation group showed significantly higher NCV compared with the control group (31.72 m/s vs. control, *p* < 0.001) and the 0 Hz group (*p* = 0.002). The 20 Hz group also exhibited increased NCV (32.27 m/s) compared with control (*p* < 0.001). In contrast, no significant difference in NCV was observed between the 200 Hz group and control (*p* = 0.564).

Latency measurements showed that the 2 Hz and 20 Hz groups had significantly shorter latencies compared with control (2 Hz: 1.23 ms, *p* = 0.021; 20 Hz: 1.18 ms, *p* = 0.014). No significant differences were observed in the 0 Hz or 200 Hz groups.

The 2 Hz group exhibited the highest CMAP amplitude (10.28 mV) and MAP area (13.05 mV·ms), both significantly greater than those of the control and other treatment groups (*p* < 0.01). The 20 Hz group also showed increased CMAP amplitude and MAP area compared with control. No significant differences were observed in the 200 Hz group compared with control.

### 2.3. Muscle Weight Recovery and Histological Evaluation of Gastrocnemius Muscle

**Figure 3:**
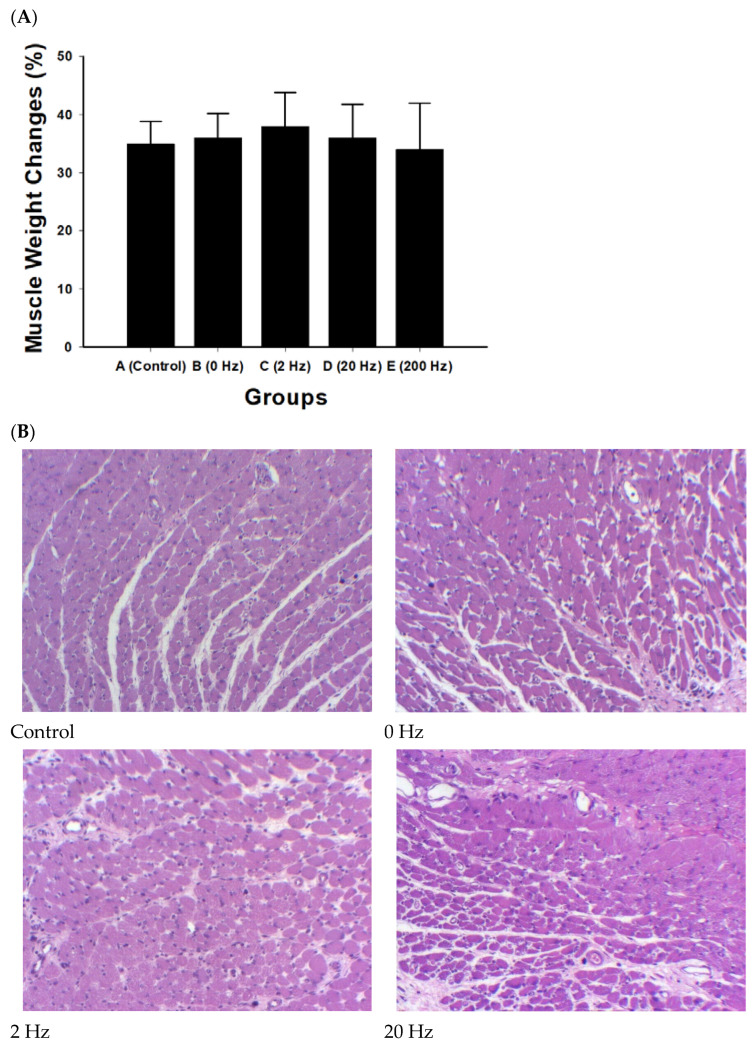

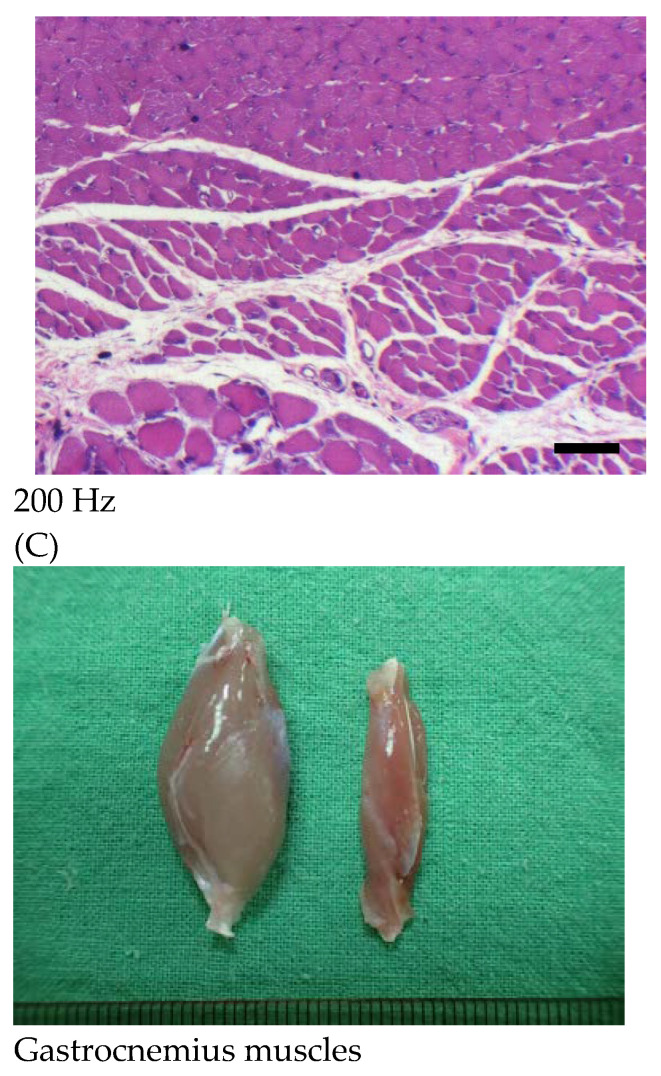
Muscle weight recovery and histological evaluation of the gastrocnemius muscle following sciatic nerve repair with BVSM nerve conduits under different ES frequencies. (**A**) Quantitative analysis of gastrocnemius muscle weight recovery expressed as a percentage relative to the contralateral normal side in the control, 0 Hz, 2 Hz, 20 Hz, and 200 Hz groups. Data are presented as mean ± SD (*n* = 10 per group). No statistically significant differences were observed among the groups. (**B**) Representative H&E-stained sections of gastrocnemius muscle from each experimental group: control, 0 Hz, 2 Hz, 20 Hz, and 200 Hz. Muscle fibers in the control group exhibited features associated with denervation, including reduced fiber size and increased interstitial spacing. Treatment groups showed generally comparable morphology with mild preservation of muscle architecture, without evident fibrosis, necrosis, or inflammatory infiltration. The black rectangle indicates the scale bar, scale bar = 50 μm. (**C**) Representative images of gastrocnemius muscles harvested from the contralateral (left) and operated (right) hind limbs, showing visible muscle atrophy following nerve injury.

Muscle weight did not differ significantly among experimental groups (all comparisons, *p* > 0.2). The 2 Hz ES group showed a higher mean value (38 ± 5.9%), although the difference was not statistically significant (Figure 3A).

Histological examination of gastrocnemius muscle sections showed generally comparable morphological features among the experimental groups. The control group exhibited reduced muscle fiber size and increased interstitial spacing.

In the treatment groups, muscle fibers were present with varying degrees of organization. No marked fibrosis, necrosis, or inflammatory infiltration was observed across groups (Figure 3B). The 2 Hz and 20 Hz groups showed relatively more compact fiber arrangement compared with control; however, these observations were not quantitatively assessed.

### 2.4. Functional Recovery Assessed by Rotarod Performance

**Figure 4:**
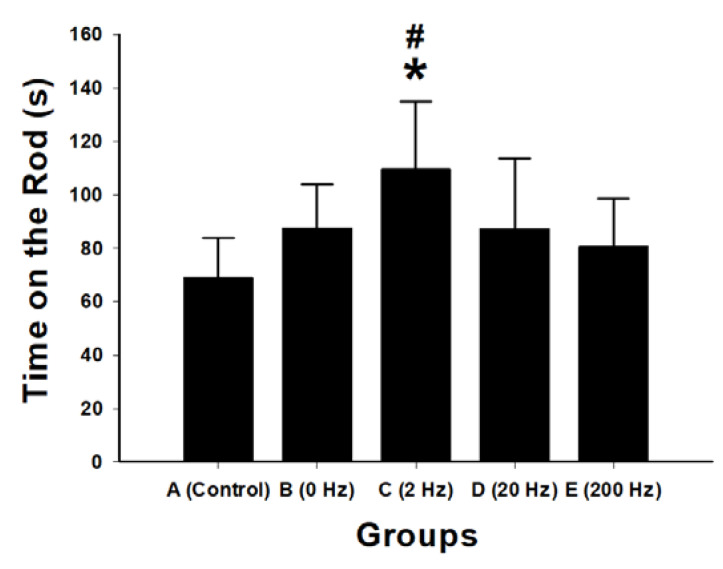
Functional motor recovery assessed by rotarod performance following sciatic nerve repair with BVSM nerve conduits under different ES frequencies. Bar graph showing the latency to fall (seconds; s) in the rotarod test for the control, 0 Hz, 2 Hz, 20 Hz, and 200 Hz groups. Data are presented as mean ± SD (*n* = 10 per group). The 2 Hz stimulation group demonstrated significantly improved motor coordination and endurance compared with the control and other stimulation groups. * *p* < 0.05 vs. control group; # *p* < 0.05 vs. 0 Hz group.

Functional motor recovery following sciatic nerve repair was evaluated using the rotarod test. The 2 Hz ES group exhibited the longest latency to fall (109.8 ± 25.3 s), which was significantly greater than that of the control group (*p* < 0.001), 0 Hz group (*p* = 0.026), 20 Hz group (*p* = 0.024), and 200 Hz group (*p* = 0.007) (Figure 4).

No significant differences were observed between the 20 Hz and 200 Hz groups compared with the 0 Hz group.

### 2.5. Sensory Recovery and Nociceptive Behavioral Outcomes

**Figure 5:**
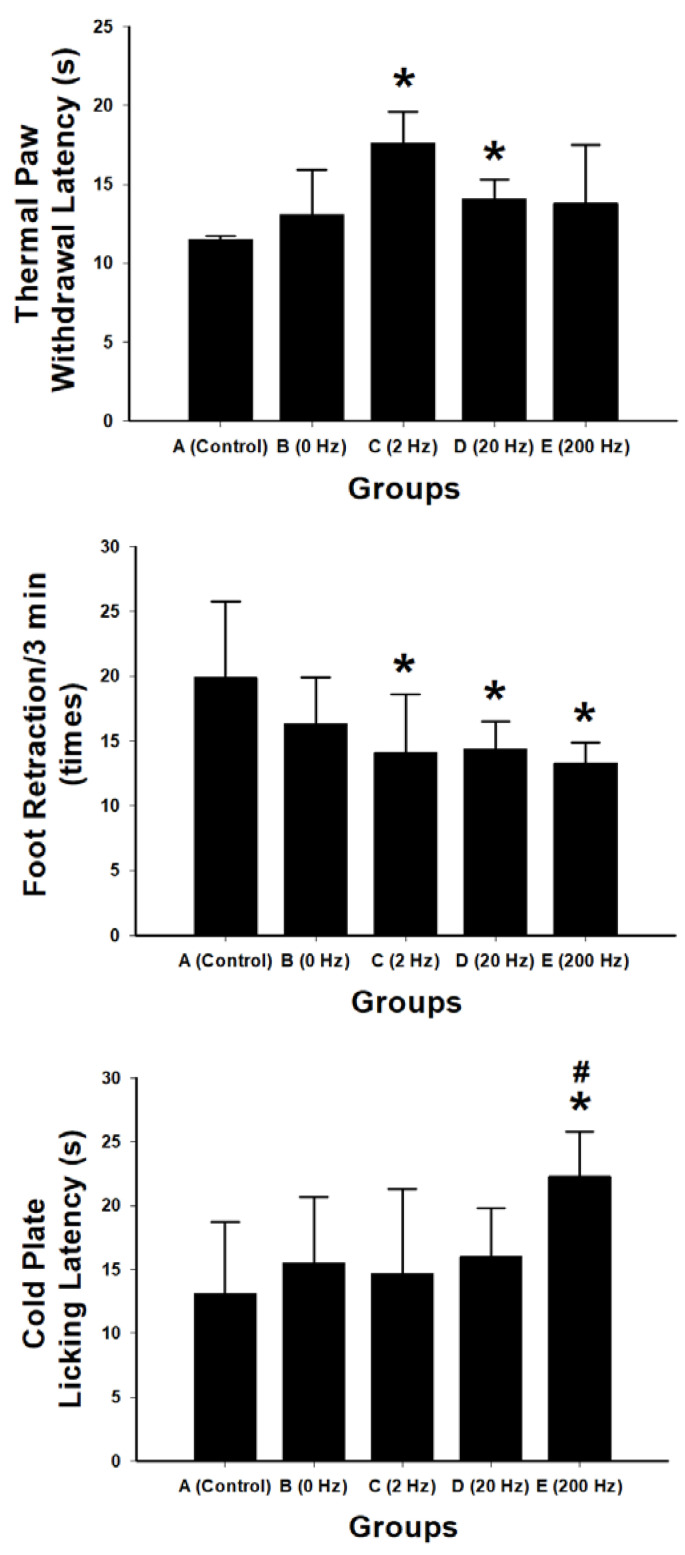
Sensory and nociceptive behavioral assessments following sciatic nerve repair with BVSM nerve conduits under different ES frequencies. Thermal paw withdrawal latency test showing the response time to a thermal stimulus in the control, 0 Hz, 2 Hz, 20 Hz, and 200 Hz groups. Spontaneous nociceptive behavior measured by the frequency of foot retractions within a 3 min observation period. Cold nociceptive sensitivity evaluated using cold plate licking latency. Data are presented as mean ± SD (*n* = 10 per group). The 2 Hz stimulation group exhibited significantly prolonged thermal withdrawal latency and reduced spontaneous nociceptive behavior compared with the control group, indicating improved sensory recovery and reduced pain-like responses. In contrast, the 200 Hz group demonstrated significantly increased cold plate licking latency, suggesting enhanced tolerance to cold nociceptive stimuli. * *p* < 0.05 vs. control group; # *p* < 0.05 vs. 2 Hz group.

Sensory recovery following sciatic nerve repair was evaluated using thermal withdrawal latency, spontaneous nociceptive behavior, and cold sensitivity testing (Figure 5).

Thermal nociceptive testing showed that the 2 Hz stimulation group exhibited the longest paw withdrawal latency (17.6 ± 2.0 s), which was significantly greater than that of the control group (*p* = 0.026) and the 20 Hz group (*p* = 0.042). No significant differences were observed between the 0 Hz or 200 Hz groups and the control group.

Spontaneous nociceptive behavior was assessed by quantifying foot retractions over a 3 min observation period. The 2 Hz group exhibited the lowest mean retraction frequency (14.1 ± 4.5), which was significantly lower than that of the control group (*p* = 0.007), the 20 Hz group (*p* = 0.026), and the 200 Hz group (*p* = 0.006). The 0 Hz group showed a reduction compared with control, although the difference was not statistically significant (*p* = 0.088). No significant differences were observed among the stimulated groups (all *p* > 0.7).

Cold nociceptive sensitivity was evaluated using cold plate licking latency. Mean licking latencies were 13.1 ± 5.6 s in the control group, 15.5 ± 5.2 s in the 0 Hz group, 14.7 ± 6.6 s in the 2 Hz group, 16.0 ± 3.8 s in the 20 Hz group, and 22.3 ± 3.5 s in the 200 Hz group. The 200 Hz group showed significantly increased latency compared with the control group (*p* = 0.004) and the 2 Hz group (*p* = 0.009). The 20 Hz group also showed a significant increase compared with the 2 Hz group (*p* = 0.029). No significant differences were observed between the 2 Hz or 0 Hz groups and control.

### 2.6. Retrograde Tracing of Functional Neuronal Reconnection

**Figure 6:**
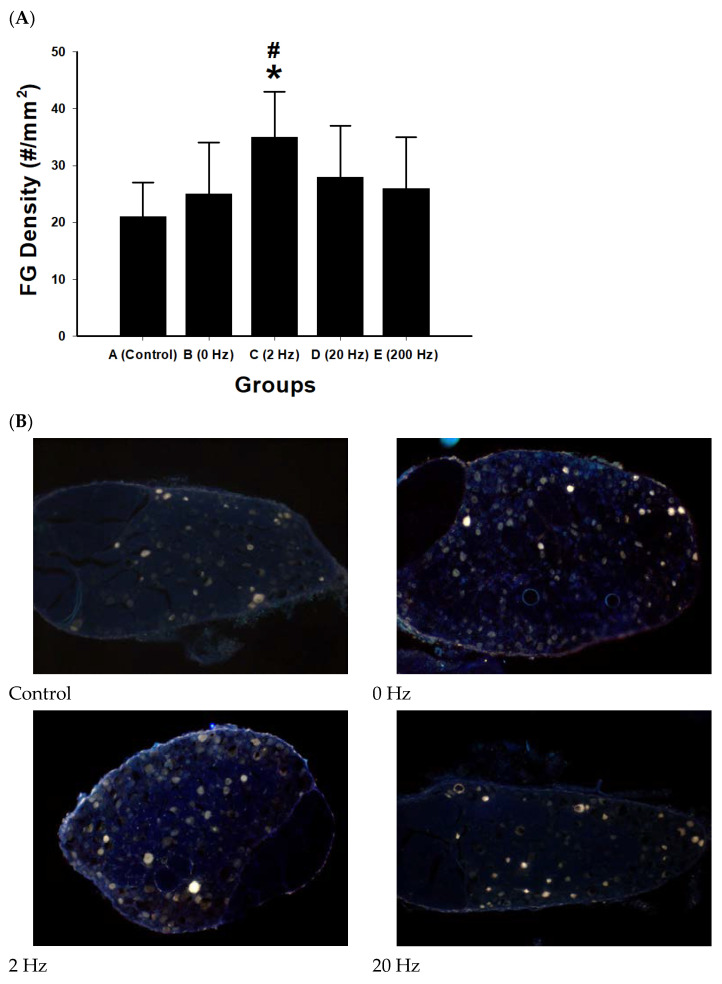

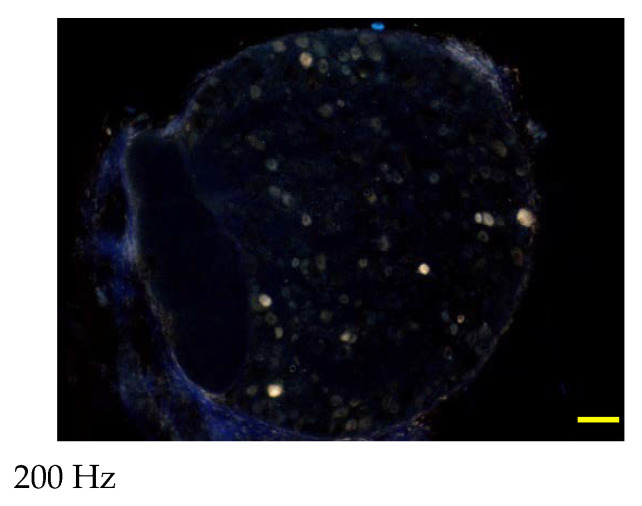
Retrograde tracing of functional neuronal reconnection following sciatic nerve repair. (**A**) Quantification of FG-labeled neurons in the spinal cord following retrograde tracing from the distal sciatic nerve. The density of FG-positive neurons (cells/mm^2^) was compared among experimental groups: control, 0 Hz, 2 Hz, 20 Hz, and 200 Hz. The 2 Hz ES group exhibited the highest density of labeled neurons, indicating enhanced neuronal reconnection. Data are presented as mean ± SD (*n* = 10 per group). * *p* < 0.05 versus control; # *p* < 0.05 versus 0 Hz group. (**B**) Representative fluorescence micrographs showing FG-labeled neurons in the corresponding experimental groups: control, 0 Hz, 2 Hz, 20 Hz, and 200 Hz. FG-positive neurons appear as bright punctate signals distributed within the spinal cord tissue. The 2 Hz stimulation group shows a visibly greater number of labeled neurons compared with other groups, indicating improved retrograde transport and functional reconnection between proximal neurons and distal targets. The yellow rectangle indicates the scale bar, scale bar = 100 μm.

FG retrograde labeling was performed to evaluate neuronal reconnection between proximal neurons and distal target tissues following sciatic nerve repair. Quantification of FG-labeled neurons showed differences among stimulation groups (Figure 6).

The 2 Hz stimulation group exhibited the highest density of FG-labeled neurons (35 ± 8 cells/mm^2^), which was significantly greater than that of the control group (*p* = 0.001) and the 0 Hz group (*p* = 0.015). The 20 Hz group showed an increase in labeled neuron density; however, this difference was not statistically significant compared with control (*p* = 0.076). No significant difference was observed between the 200 Hz group and control.

### 2.7. Macrophage Infiltration and Inflammatory Response

**Figure 7:**
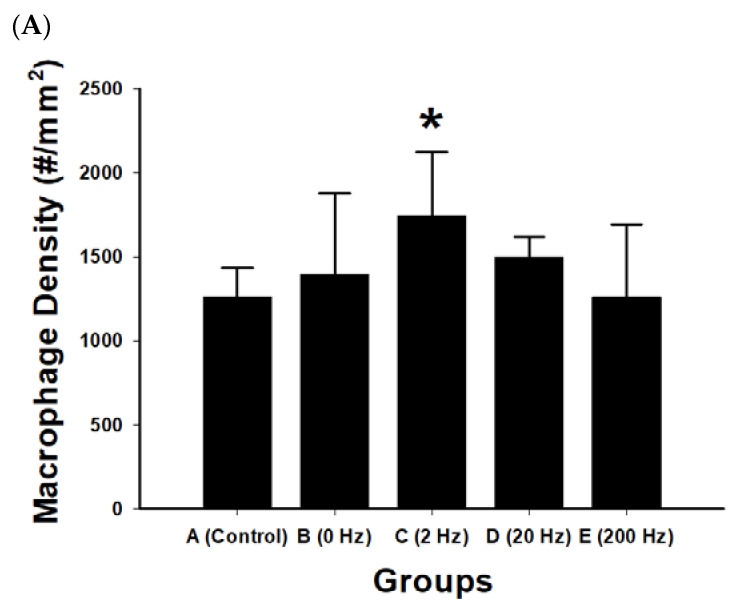

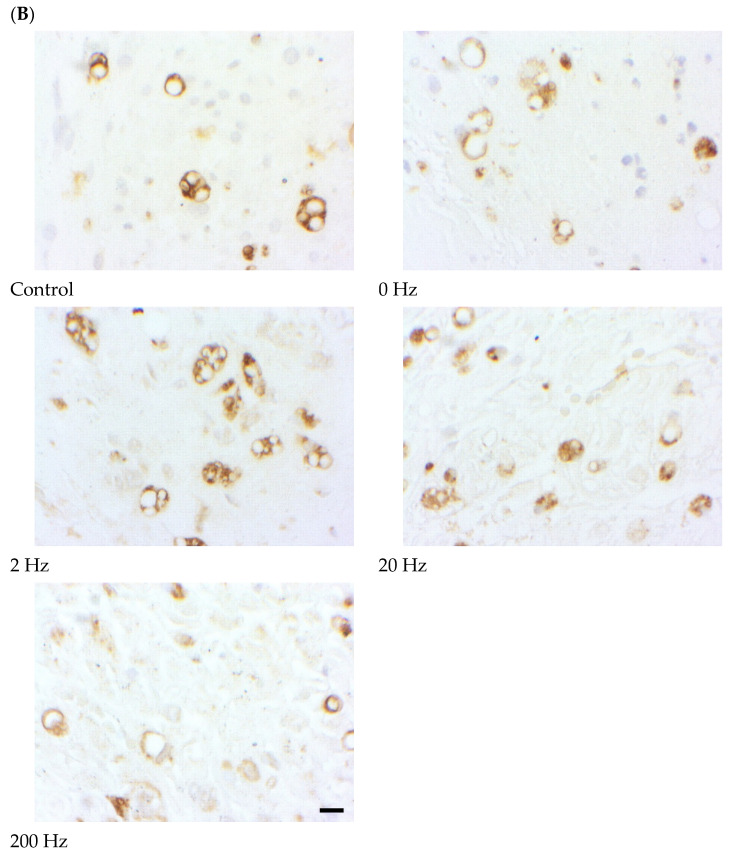
Macrophage infiltration in regenerated nerve tissue following ES. (**A**) Quantitative analysis of macrophage density (cells/mm^2^) in regenerated sciatic nerve tissue across experimental groups: control, 0 Hz, 2 Hz, 20 Hz, and 200 Hz. The 2 Hz ES group exhibited the highest macrophage density, which was significantly greater than that of the control group. Data are presented as mean ± SD (*n* = 10 per group). * *p* < 0.05 versus control. (**B**) Representative immunohistochemical images showing macrophage distribution within regenerated nerve tissue in each experimental group: control), 0 Hz, 2 Hz, 20 Hz, and 200 Hz. Macrophages are indicated by brown-stained cells within the nerve tissue. Increased macrophage presence is observed in the 2 Hz stimulation group compared with other groups. The black rectangle indicates the scale bar, scale bar = 50 μm.

Macrophage density within regenerated nerve tissue was quantified to evaluate inflammatory cell presence under different electrical stimulation frequencies. Differences among groups were observed (Figure 7).

The 2 Hz stimulation group exhibited the highest macrophage density (1744.2 ± 376.1 cells/mm^2^), which was significantly greater than that of the control group (*p* = 0.021). In contrast, the 200 Hz group showed the lowest macrophage density (1259.9 ± 433.4 cells/mm^2^), which was significantly lower than that of the 2 Hz group (*p* = 0.020).

No statistically significant differences were observed among other pairwise comparisons (all *p* > 0.05), including comparisons between the control and 0 Hz or 20 Hz groups.

### 2.8. CGRP Expression and Peptidergic Sensory Activation

**Figure 8:**
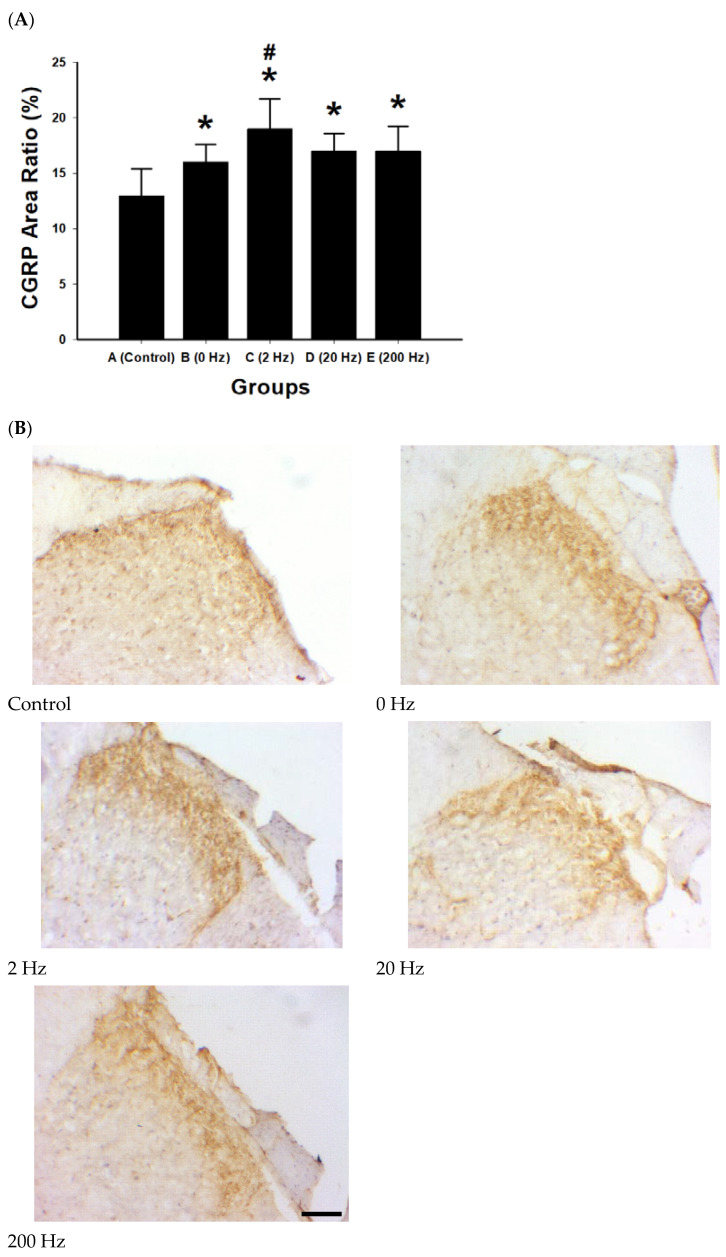
CGRP expression and peptidergic sensory activation following ES. (**A**) Quantitative analysis of CGRP immunoreactivity expressed as area ratio (%) in regenerated nerve tissue across experimental groups: control), 0 Hz, 2 Hz, 20 Hz, and 200 Hz. All electrical stimulation (ES) groups exhibited significantly higher CGRP expression compared with the control group. The 2 Hz stimulation group showed the highest CGRP area ratio and was significantly greater than the 0 Hz group. Data are presented as mean ± SD (*n* = 10 per group). * *p* < 0.05 versus control; # *p* < 0.05 versus 0 Hz group. (**B**) Representative immunohistochemical images of CGRP staining in regenerated nerve tissue for each experimental group: control, 0 Hz, 2 Hz, 20 Hz, and 200 Hz. CGRP-positive areas appear as brown immunoreactive staining within the tissue, indicating activation of peptidergic sensory pathways. The 2 Hz stimulation group demonstrates more extensive CGRP immunoreactivity compared with other groups. The black rectangle indicates the scale bar, scale bar = 50 μm.

CGRP immunoreactivity was quantified as area ratio (%) to evaluate changes under different electrical stimulation frequencies (Figure 8). A stimulation-dependent increase in CGRP expression was observed.

The 2 Hz group exhibited the highest CGRP area ratio (19% ± 2.7%), followed by the 20 Hz and 200 Hz groups (both approximately 17%), whereas the control group showed the lowest expression level (13%). All stimulation groups showed significantly increased CGRP expression compared with control (all *p* < 0.01).

Among stimulated groups, the 2 Hz condition showed a significantly higher CGRP area ratio compared with the 0 Hz group (*p* = 0.038). No significant differences were observed between the 20 Hz and 200 Hz groups (both *p* > 0.05).

### 2.9. Quantitative Morphometric Analysis of Axonal Regeneration

**Figure 9:**
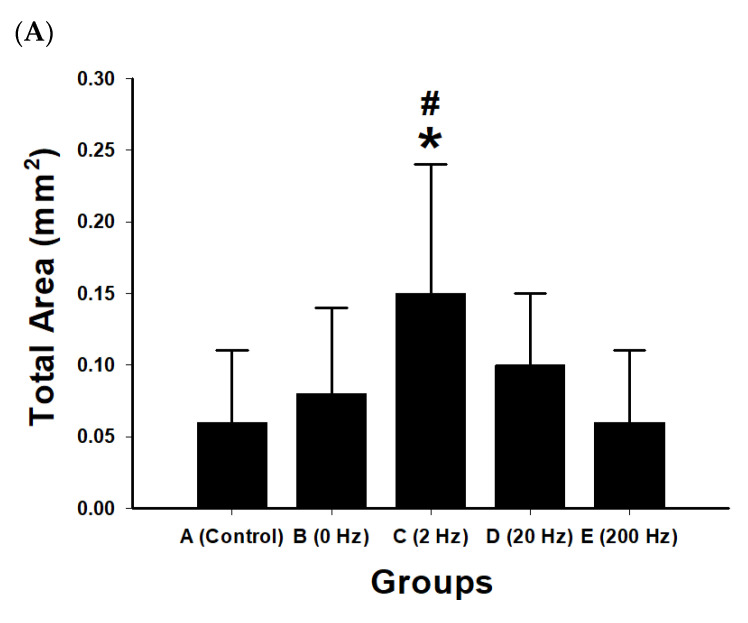

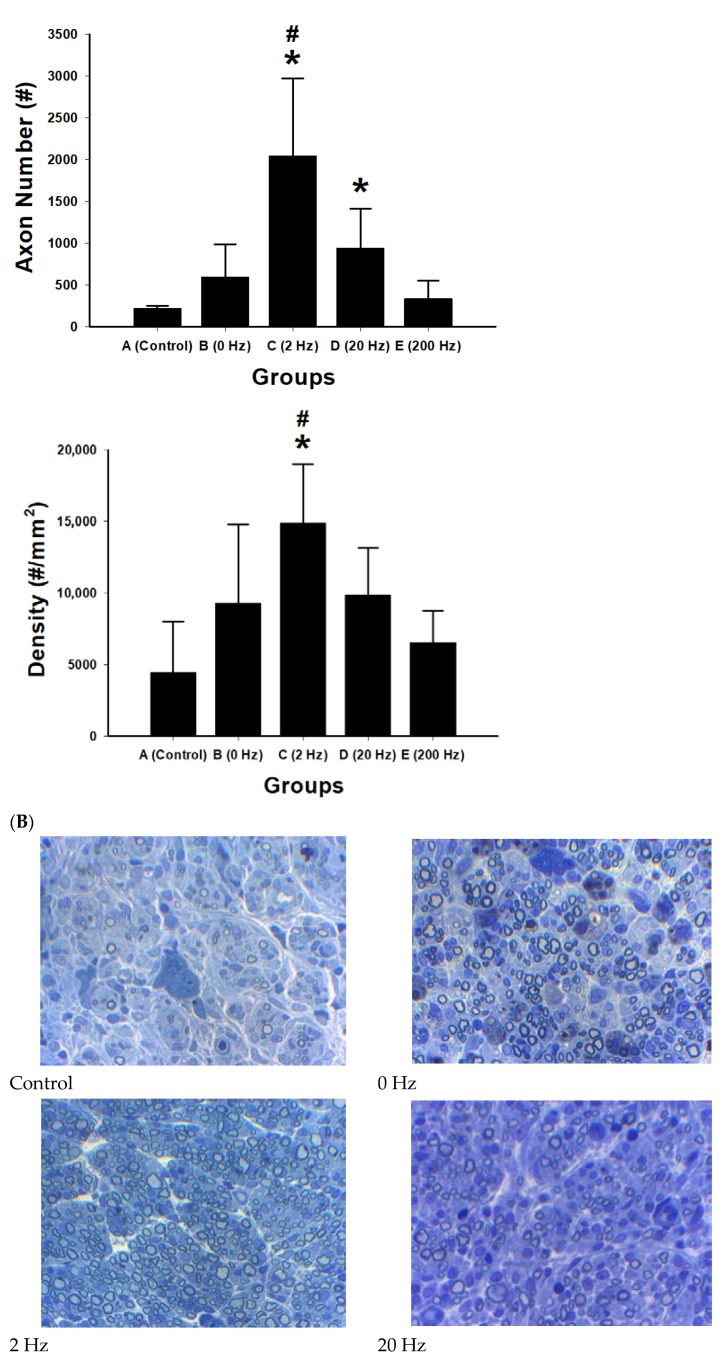

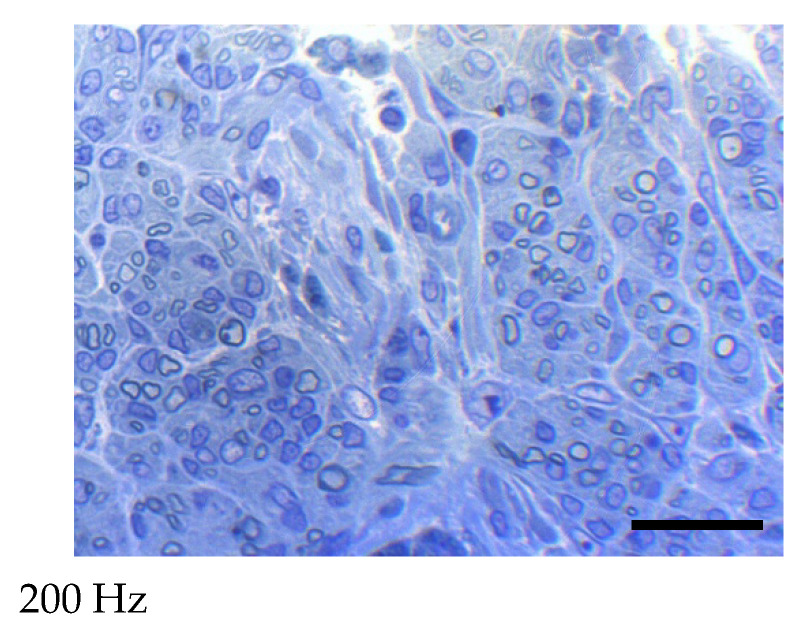
Morphometric and histological evaluation of axonal regeneration under different ES frequencies. (**A**) Quantitative morphometric analysis of regenerated axons in five experimental groups: control, 0 Hz, 2 Hz, 20 Hz, and 200 Hz. Bar graphs show total axonal area (mm^2^), axon number, and axon density (axons/mm^2^). Data are presented as mean ± SD (*n* = 10 per group). The 2 Hz stimulation group exhibited the greatest regenerative response, showing the largest total axonal area, highest axon number, and greatest axonal density among all groups. Moderate increases were observed in the 0 Hz and 20 Hz groups, whereas the 200 Hz group showed reduced regenerative indices compared with the 2 Hz group. * *p* < 0.05 versus control; # *p* < 0.05 versus 200 Hz. (**B**) Representative cross-sectional micrographs of regenerated nerve fibers stained with toluidine blue in each group: control, 0 Hz, 2 Hz, 20 Hz, and 200 Hz. The 2 Hz group demonstrates a higher density of myelinated axons and more organized nerve fiber architecture compared with other groups. The control group shows sparse axonal regeneration, while higher-frequency stimulation groups display comparatively reduced axonal density. The black rectangle indicates the scale bar, scale bar = 20 μm.

Quantitative morphometric analysis of regenerated nerve sections showed differences among the experimental groups in total axonal area, axon number, and axonal density (Figure 9).

The 2 Hz group showed the largest total axonal area (0.15 mm^2^), the highest axon number, and the greatest axonal density (14,883 axons/mm^2^). The control group showed the lowest values for these parameters. The 0 Hz and 20 Hz groups showed higher values than the control group, whereas the 200 Hz group showed lower values than the 2 Hz group.

Compared with the control group, the 2 Hz group showed significant increases in total axonal area (*p* = 0.045), axon number (*p* < 0.001), and axonal density (*p* = 0.001). The 20 Hz group showed a significant increase in axon number compared with control (*p* = 0.026), whereas differences in total axonal area and axonal density were not statistically significant.

Comparisons between the 2 Hz and 200 Hz groups showed significant differences in total axonal area (*p* = 0.027), axon number (*p* < 0.001), and axonal density (*p* = 0.004). No significant differences were observed among the control, 0 Hz, 20 Hz, and 200 Hz groups in the remaining pairwise comparisons.

### 2.10. Expression of Growth Factors and Inflammatory Cytokines

**Figure 10:**
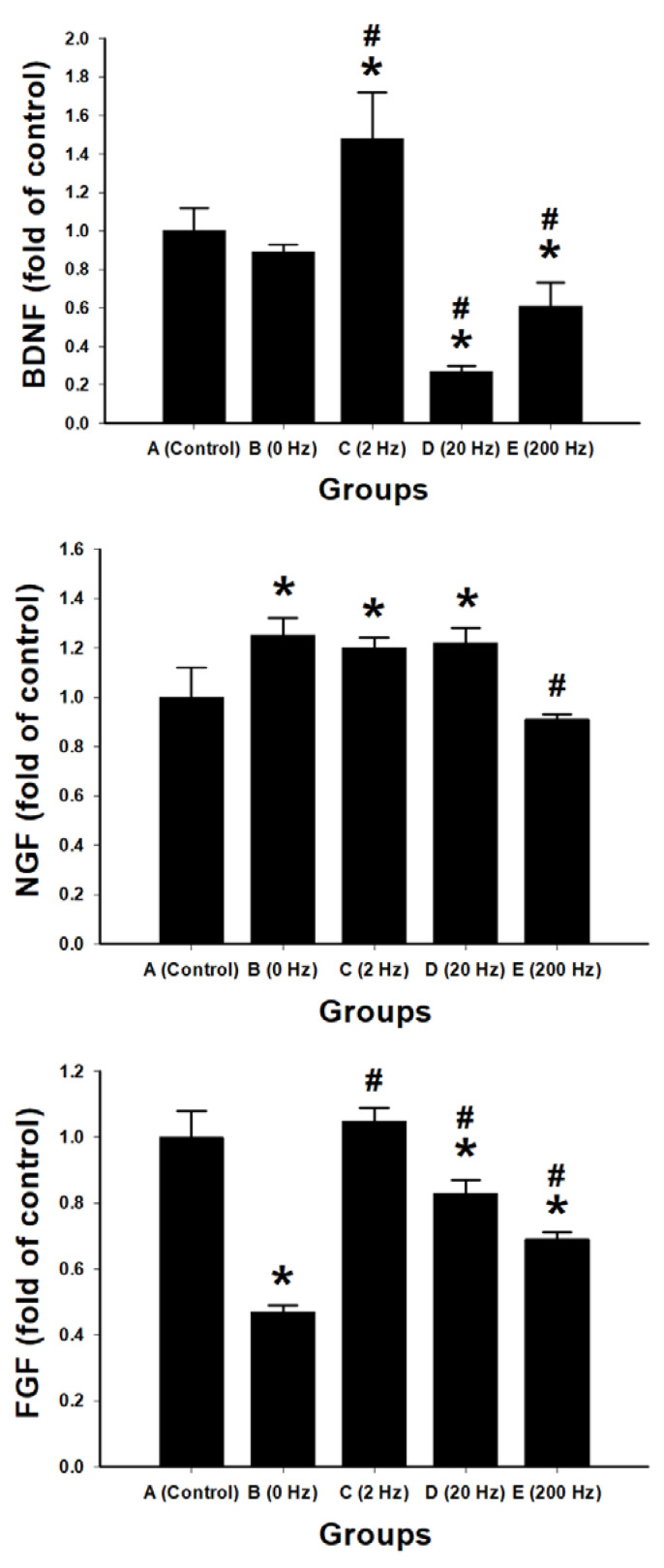

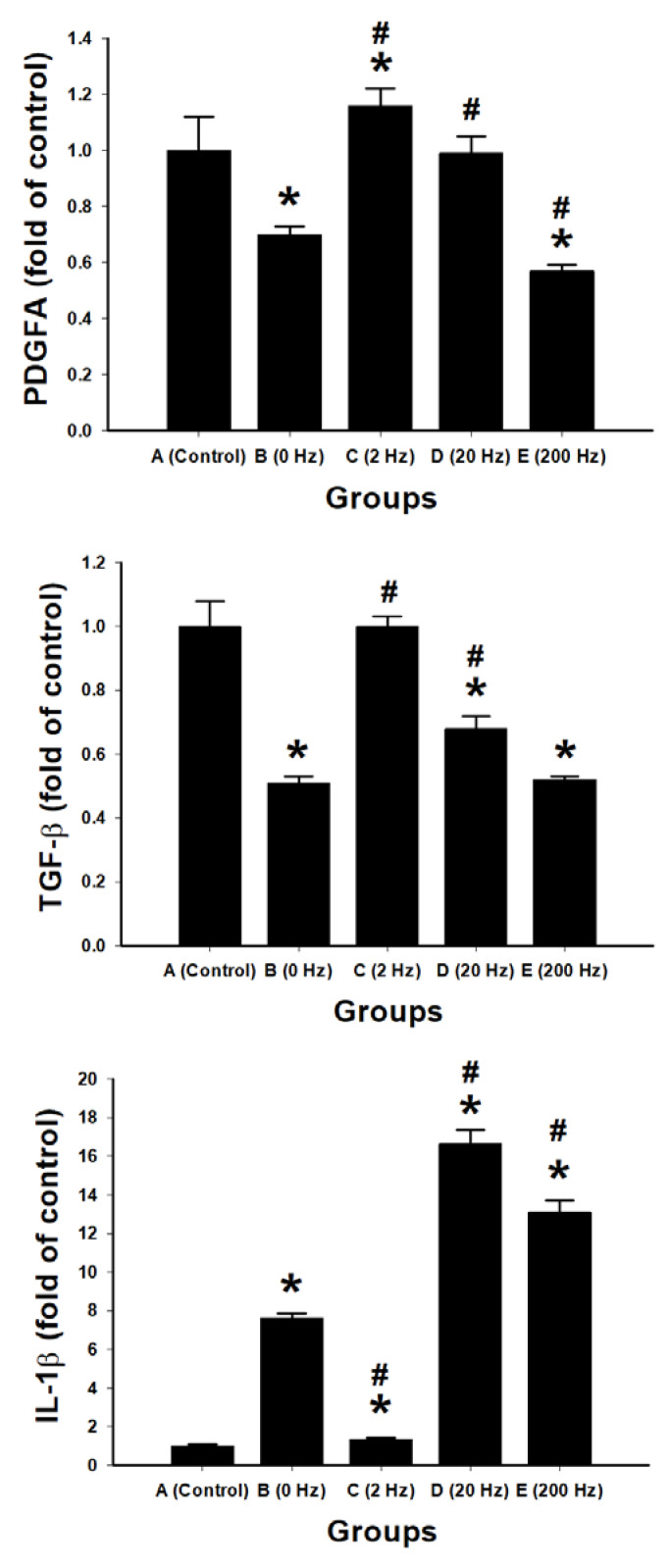

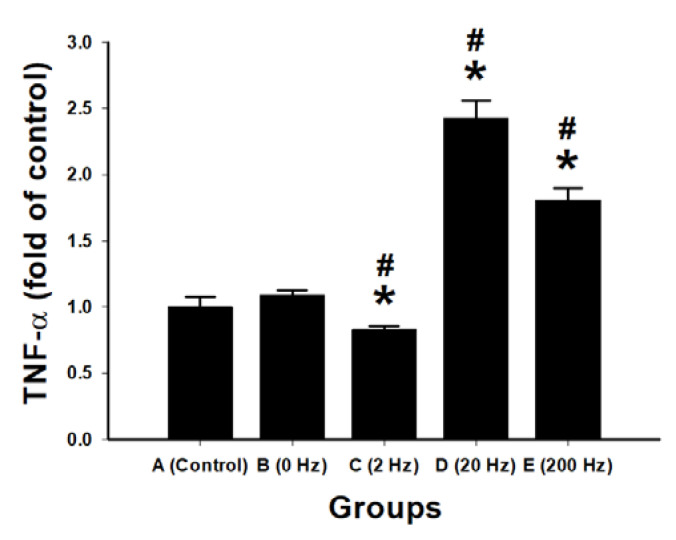
Frequency-dependent regulation of neurotrophic factors and inflammatory cytokines in regenerated nerve tissue. Quantitative RT-PCR analysis of gene expression for neurotrophic factors (BDNF, NGF, FGF, PDGFA, and TGF-β) and inflammatory cytokines (IL-1β and TNF-α) in regenerated nerve tissue following ES at different frequencies. Data are presented as fold change relative to the control group. Experimental groups include: control, 0 Hz, 2 Hz, 20 Hz, and 200 Hz. Data are expressed as mean ± SD (*n* = 10 per group). * *p* < 0.05 vs. control group; # *p* < 0.05 vs. 0 Hz group.

Quantitative RT-PCR was performed to assess the expression of neurotrophic factors (BDNF, NGF, FGF, PDGFA, TGF-β) and inflammatory cytokines (IL-1β, TNF-α) in regenerated nerve tissues (Figure 10).

In the 2 Hz group, BDNF expression was significantly increased (1.48 ± 0.24-fold vs. control, *p* = 0.001). PDGFA expression was also significantly increased (1.16 ± 0.06-fold vs. control, *p* = 0.015). NGF expression was elevated (1.20 ± 0.04-fold vs. control, *p* = 0.007). FGF expression (1.05 ± 0.04-fold) and TGF-β expression were comparable to control.

In the 20 Hz group, NGF expression was increased (1.22 ± 0.06-fold vs. control, *p* = 0.004), whereas BDNF expression was decreased (0.27 ± 0.03-fold vs. control, *p* < 0.001). TGF-β expression was also significantly reduced compared with control (*p* < 0.001).

In the 200 Hz group, expression levels of BDNF (0.61 ± 0.12-fold), PDGFA (0.57 ± 0.02-fold), FGF (0.69 ± 0.02-fold), and TGF-β (0.52 ± 0.01-fold) were significantly lower than those in the control group (*p* < 0.01).

In the 0 Hz group, FGF and PDGFA expression levels were significantly lower than those in the control group (both *p* < 0.001).

IL-1β expression was significantly increased in the 20 Hz (16.62 ± 0.76-fold), 200 Hz (13.10 ± 0.60-fold), and 0 Hz (7.62 ± 0.26-fold) groups compared with control (all *p* < 0.001). The 2 Hz group showed a lower level of IL-1β expression (1.34 ± 0.08-fold), which was significantly lower than that in the other stimulation groups (*p* < 0.001).

TNF-α expression was significantly increased in the 20 Hz (2.43 ± 0.13-fold) and 200 Hz (1.81 ± 0.09-fold) groups compared with control (*p* < 0.001). The 2 Hz group showed a lower TNF-α level (0.83 ± 0.03-fold), which was significantly lower than that in the 20 Hz and 200 Hz groups.

## 3. Discussion

This study demonstrates that the combination of BVSM-crosslinked conduits and ES provides both mechanical guidance and bioelectrical modulation—two fundamental components of the native regenerative niche. BVSM networks offer tunable elasticity and chemical stability comparable to native nerve tissue, while their hydrophilicity maintains a permissive microenvironment for cellular infiltration and migration. Previous reports indicate that ES enhances this regenerative niche by aligning Schwann cells, polarizing growth cones, and modulating the secretion of neurotrophic factors such as IGF-1, BDNF, and GDNF [14,15,16]. Together, these structural and biochemical cues synergistically accelerate axonal elongation, remyelination, and synaptic reconnection.

Importantly, the BVSM conduits withstood repeated electrical stimulation without visible degradation or structural collapse, demonstrating polymer stability under mild electrochemical conditions. Unlike aldehyde-based crosslinkers such as glutaraldehyde, BVSM forms stable thioether linkages under mild reaction conditions, thereby avoiding cytotoxic residues and preserving gelatin’s intrinsic bioactivity [17]. These characteristics likely contributed to the minimal inflammatory response and favorable host–implant interaction observed in vivo. Structural stability is critical for maintaining aligned regenerative pathways and preventing scar infiltration, both prerequisites for effective axonal guidance. Collectively, these findings validate BVSM as a promising crosslinker for bioelectronic nerve repair devices requiring mechanical resilience and long-term biocompatibility.

Among all tested stimulation paradigms, the present study consistently demonstrates a frequency-dependent effect, in which low-frequency stimulation at 2 Hz produces the most favorable overall regenerative outcomes across structural, functional, and molecular levels. Specifically, 2 Hz stimulation resulted in significantly greater axon number, axonal density, and regenerated nerve area, together with improved electrophysiological performance, enhanced motor coordination, and superior sensory recovery compared with unstimulated or high-frequency conditions. In contrast, higher frequencies, particularly 200 Hz, did not improve these parameters and in some cases showed reduced values relative to the 2 Hz condition [18,19,20].

The bell-shaped frequency–response pattern observed here is consistent with previous studies demonstrating that peripheral nerve regeneration is optimized within a specific stimulation range. Geremia et al. reported that 20 Hz stimulation accelerates motor axon outgrowth and increases expression of GAP-43 and BDNF after sciatic nerve injury [21], while Al-Majed et al. demonstrated that brief 20 Hz stimulation enhances target reinnervation and recovery [22]. Similar benefits have been reported in conduit-based repair models [23,24]. However, the present results extend these findings by showing that sustained low-frequency stimulation at 2 Hz provides superior and more consistent benefits across multiple outcome measures, rather than intermediate frequencies such as 20 Hz.

This frequency-dependent divergence is further supported by the molecular data. The 2 Hz condition was associated with increased expression of neurotrophic factors, including BDNF, PDGFA, and NGF, whereas higher frequencies (20 and 200 Hz) were associated with reduced expression of several growth factors and marked elevation of pro-inflammatory cytokines such as IL-1β and TNF-α. These findings suggest that different stimulation frequencies differentially regulate the balance between neurotrophic signaling and inflammatory activation, which is a critical determinant of regenerative efficiency.

One possible explanation is that low-frequency stimulation more closely mimics physiological neuronal firing patterns, thereby promoting periodic depolarization and calcium oscillations that support cytoskeletal remodeling, vesicular transport, and gene transcription. In contrast, high-frequency stimulation may induce excessive ionic flux, intracellular calcium overload, and metabolic stress, which can disrupt signaling pathways required for axonal growth and survival. This interpretation is consistent with previous reports indicating that overstimulation may impair regeneration through neuronal fatigue and dysregulated intracellular signaling [18,19,20].

In the present study, electrophysiological assessments showed that 2 Hz stimulation yielded the highest nerve conduction velocity and MAP amplitude with significantly shortened latencies compared with other groups. These findings agree with prior reports that low-frequency ES accelerates axonal elongation, promotes Schwann-cell proliferation, and upregulates neurotrophic factors [12,25,26]. However, our findings indicate that sustained low-frequency stimulation at 2 Hz provides superior overall outcomes. This may be explained by the fact that periodic depolarization at low frequency more closely mimics physiological firing patterns, stabilizing membrane potentials and promoting calcium oscillations required for cytoskeletal remodeling and vesicular trafficking [27]. In contrast, high-frequency (200 Hz or more) stimulation did not improve conduction, possibly due to ionic overload or metabolic stress that interferes with regenerative signaling [28,29].

Electrophysiological findings further support the superiority of 2 Hz stimulation. This group demonstrated the highest nerve conduction velocity, increased MAP amplitude, and shortened latency, indicating enhanced axonal integrity and remyelination. Behavioral outcomes paralleled these improvements: animals receiving 2 Hz stimulation showed superior rotarod performance and longer thermal withdrawal latency, reflecting improved motor coordination and sensory recovery. Retrograde Fluoro-Gold labeling confirmed greater neuronal reconnection in the 2 Hz group, demonstrating that structural regeneration translated into functional reinnervation.

Importantly, integration of morphometric, electrophysiological, behavioral, and retrograde tracing results indicates that the effects of 2 Hz stimulation are not limited to a single domain but reflect coordinated improvements across multiple levels of nerve regeneration, from axonal growth to functional recovery.

Interestingly, 200 Hz stimulation improved cold nociceptive responses but did not enhance overall structural or motor recovery. This suggests selective modulation of specific sensory pathways, potentially involving Aδ fibers or central nociceptive circuits, rather than broad enhancement of axonal regeneration. Similar frequency-dependent dissociation between regenerative and sensory-modulatory effects has been reported previously [30,31]. Thus, lower frequencies appear to favor structural repair and reconnection, whereas higher frequencies may predominantly modulate sensory processing.

Macrophage infiltration exhibited a frequency-dependent pattern, with the highest density observed in the 2 Hz group. Controlled macrophage recruitment is essential for debris clearance and for secretion of cytokines that stimulate Schwann cell differentiation and angiogenesis [32]. The enhanced macrophage presence under 2 Hz stimulation likely facilitated a timely transition from inflammation to regeneration. Concurrently, CGRP expression was significantly elevated under low-frequency stimulation. As CGRP participates in vasodilation and neurotrophic signaling, its upregulation may reflect increased neuronal activation and improved trophic support to regenerating fibers [33,34]. Together, these findings suggest that 2 Hz ES promotes a coordinated neuroimmune and neurochemical environment conducive to regeneration rather than chronic inflammation.

The gene expression data further support this neuroimmune coordination. While high-frequency stimulation induced pronounced upregulation of IL-1β and TNF-α, the 2 Hz condition maintained relatively low levels of these cytokines, suggesting a more controlled inflammatory response.

Although macrophage phenotypes were not directly assessed in this study, the observed increase in macrophage density under 2 Hz stimulation, together with reduced expression of pro-inflammatory cytokines, suggests a shift toward a pro-regenerative immune microenvironment. It is well established that M2-like macrophages contribute to debris clearance, angiogenesis, and secretion of trophic factors that support axonal regeneration, whereas prolonged M1 activation is associated with sustained inflammation and impaired healing. Therefore, low-frequency electrical stimulation may promote a favorable balance between inflammatory and regenerative responses, potentially through modulation of macrophage polarization.

Taken together, the present findings support a model in which low-frequency electrical stimulation (2 Hz) optimizes peripheral nerve regeneration by simultaneously enhancing neurotrophic signaling and limiting excessive inflammatory activation, thereby creating a permissive microenvironment for axonal growth and functional recovery. In contrast, higher-frequency stimulation appears to shift this balance toward inflammatory activation and reduced trophic support, which may compromise regenerative efficiency.

Since the 4-week evaluation revealed significant improvements, longer-term studies are required to assess complete remyelination, sustained muscle reinnervation, and eventual biodegradation of BVSM conduits. Future investigations should also explore adaptive or feedback-controlled stimulation paradigms that adjust frequency and amplitude according to regenerative stage, potentially maximizing therapeutic efficacy.

Although autologous nerve grafting remains the clinical gold standard for peripheral nerve repair, it was not included as a comparison group in the present study. The primary aim of this study was to investigate the frequency-dependent effects of electrical stimulation within BVSM-based nerve guidance conduits. Future studies incorporating autograft controls are warranted to further benchmark the performance of this approach.

## 4. Materials and Methods

### 4.1. Fabrication of Gelatin-BVSM Conduits

A 10% (*w*/*w*) gelatin solution was prepared by dissolving gelatin (Sigma, #G2500, St. Louis, MO, USA) in 0.2 M Na_2_HPO_4_ buffer under magnetic stirring at 60 °C. A silicone rubber tube (outer diameter: 1.96 mm; Helix Medical, Inc., Carpinteria, CA, USA) was used as a mandrel and vertically dipped into the gelatin solution at a constant rate, where it was maintained for 30 s. This coating procedure was repeated nine times to achieve a conduit with an approximate wall thickness of 400 µm. The coated mandrel was air-dried for 1 h and subsequently immersed in a 0.3% (*w*/*w*) BVSM (Tokyo Chemical Industry, Tokyo, Japan) solution for 24 h to facilitate cross-linking. Following cross-linking, the gelatin-BVSM conduits were rinsed three times with 95% ethanol and air-dried for seven days. The dried conduits were carefully removed from the mandrel and sectioned into 15-mm lengths. To improve nerve fixation, small holes were drilled at both ends of each conduit. Finally, the conduits were sterilized using γ-irradiation at 25 kGy before subsequent cell culture and animal implantation experiments.

### 4.2. Gelatin-BVSM Conduit Implantation

In vivo implantation studies were conducted on fifty adult female Sprague-Dawley rats, which were randomly assigned to five experimental groups with an implantation period of 4 weeks (*n* = 10 per group). All surgical procedures were performed under general inhalational anesthesia using isoflurane (AErrane^®^, Baxter, Deerfield, IL, USA). Following a skin incision along the right thigh, the fascia and underlying muscle layers were gently separated by blunt dissection to expose the sciatic nerve. The nerve was transected to create proximal and distal stumps, which were inserted 2.5 mm into each end of a gelatin-BVSM conduit and secured with a single 9-0 nylon suture through the epineurium and the conduit wall, resulting in a 10 mm interstump gap. The muscle layer was reapproximated with 4-0 chromic gut sutures, and the skin was closed with 2-0 silk sutures. Postoperatively, animals were housed under controlled environmental conditions (22 °C, 45% relative humidity, 12 h light/dark cycle) with free access to food and water.

### 4.3. Animal Ethic and Electrical Stimulation Protocols

This study was conducted in accordance with the Guide for the Care and Use of Laboratory Animals (National Academies Press, Washington, DC, USA). All procedures were approved by the Institutional Animal Care and Use Committee (IACUC) of China Medical University, Taichung, Taiwan (Approval Number: CMUIACUC-2024-203, Date: 14 February 2024).

In brief, animals were secured in a small cage, and their stretched right leg and paw were held in place by rubber tapes. One stainless steel needle electrode (0.35 mm outer diameter, 12 mm length) connected to the negative wick (cathode) of a stimulator (Trio 300; Ito, Tokyo, Japan) was inserted aseptically into the lateral aspect of the knee, and the anode was positioned around the site of the hip joint. The positive and negative stimulating sites were near the proximal and distal ends of the implanted gelatin-BVSM conduits, respectively. The depth of insertion varied from 1 to 1.5 cm according to the thickness of skin and fatty tissues. The stimulation was applied to the animals for 15 min every other day beginning a week after the nerve repair, to avoid loosening the suture line which might also cause inflammation. The animals were divided into five groups. Group A (*n* = 10), the controls, normal animals received empty gelatin-BVSM conduits only. Groups B–E (*n* = 10 for each group), animals received a treatment of electrical stimulation of 1 mA at frequencies of 0, 2, 20, and 200 Hz, respectively, after their injured nerves were bridged with the gelatin-BVSM conduits.

### 4.4. Thermal Hyperalgesia

After 4 weeks of recovery, all the animals were tested for thermal nociceptive sensitivity using a Hargreaves analgesia meter (IITC Life Sciences, SERIES8, Model 390G, Woodland Hills, CA, USA) to measure heat-induced pain responses. A focused radiant heat source (40 °C) located beneath a glass floor was directed at the plantar surface of the rat’s hind paw. The withdrawal latency, i.e., the time taken for the rat to withdraw its paw, was measured using a stopwatch. A cut-off time of 30 s was set to prevent tissue damage. Withdrawal latency values were compared among experimental groups.

### 4.5. Cold Allodynia

Cold sensitivity was assessed using a hot/cold plate system (Panlab, Harvard Apparatus, Holliston, MA, USA). The cold plate was pre-cooled to 4 °C, and the rat was placed inside an acrylic chamber on the plate. The number of hind paw withdrawals or lifts within a 3 min test period was recorded, along with the latency to the first paw lift, representing the cold response threshold.

### 4.6. Motor Coordination Test

Motor coordination was evaluated using an accelerating rotarod device (Rotamex Rotarod, Columbus Instruments, Columbus, OH, USA). Rats were placed on a rotating rod that started at 4 rpm, with the speed increasing by 2.5 rpm every 10 s. The time each rat remained on the rod and the maximum speed tolerated before falling were recorded as indices of motor coordination and balance.

### 4.7. Electrophysiological Analysis

At the designated postoperative intervals, all animals were re-anesthetized, and the regenerated sciatic nerves were re-exposed for electrophysiological assessment. ES was applied using stainless-steel monopolar needle electrodes, with the cathode positioned 5 mm proximal to the original transection site and the anode placed 3 mm further proximally. Muscle action potentials (MAPs) were recorded from the gastrocnemius muscle using micro-needle recording electrodes. A Biopac data acquisition system (Biopac Systems, Inc., Goleta, CA, USA) was used to measure and analyze the latency, amplitude, and nerve conduction velocity (NCV) of the evoked MAPs. Latency was defined as the interval from the onset of stimulation to the initial negative deflection of the recorded potential. The amplitude and area under the curve were determined from the baseline to the maximal negative peak. To calculate NCV, the sciatic nerve was stimulated proximally and distally to the implanted conduit, and the conduction velocity was derived by dividing the distance between the stimulation sites by the latency difference between the two responses.

### 4.8. Retrograde Labeling with Fluorogold

Retrograde labeling of regenerated neurons was performed using Fluorogold (FG; Fluorochrome, Denver, CO, USA) according to previously established protocols [35]. Briefly, a 2% FG solution was prepared by dissolving the fluorescent tracer in distilled water and stored at 4 °C in the dark until use. Following completion of the electrophysiological recordings, 2% FG was injected into both the common peroneal and posterior tibial nerves using a Hamilton microsyringe. Five days after tracer injection, the animals were transcardially perfused with 200 mL of 0.9% saline followed by 4% paraformaldehyde (PFA) in 0.1 M phosphate-buffered saline (PBS, pH 7.4) at 4 °C. The ipsilateral L4 and L5 DRGs were carefully dissected, post-fixed overnight in 4% PFA, and subsequently cryoprotected by immersion in 30% phosphate-buffered sucrose solution overnight. Longitudinal cryosections of the DRGs (40 μm thick) were obtained using a cryostat and air-dried for 30 min before mounting. The labeled neuronal profiles were examined under an ultraviolet fluorescence microscope (Olympus CKX41, Tokyo, Japan), and images were captured for quantitative analysis.

### 4.9. Histological Processing

For histological examination, animals were transcardially perfused as described previously [36]. The L4 segment of the spinal cord was dissected and post-fixed in 4% PFA for 4 h. Tissues were cryoprotected by immersion in 30% sucrose at 4 °C overnight and subsequently embedded in optimal cutting temperature compound. The samples were frozen at −20 °C and sectioned at a thickness of 18 μm using a cryostat. Sections were mounted on poly-L-lysine–coated glass slides for further immunohistochemical analysis. Immunohistochemistry was performed using a two-step protocol following the manufacturer’s instructions (Novolink Polymer Detection System, Novocastra, Buffalo Grove, IL, USA). Briefly, sections were incubated in 0.3% hydrogen peroxide to quench endogenous peroxidase activity, followed by Protein Block solution (RE7102; Novocastra) to minimize non-specific binding. Samples were then sequentially treated with anti-calcitonin gene-related peptide (CGRP) primary antibody (1:1000; Calbiochem, San Diego, CA, USA), Post Primary Block (RE7111; Novocastra), and Novolink Polymer secondary antibody (RE7112; Novocastra). Diaminobenzidine was used as the chromogen, and hematoxylin was applied for counterstaining. For morphological assessment of regenerated nerves, mid-sections of the sciatic nerve were excised and fixed in 2.5% glutaraldehyde, post-fixed in 0.5% osmium tetroxide, dehydrated, and embedded in Spurr’s resin. Semi-thin sections (2 μm) were cut using a Leica EM UC6 ultramicrotome (Leica Biosystems, Wetzlar, Germany), stained with toluidine blue, and examined under a light microscope (Olympus IX70, Tokyo, Japan).

### 4.10. Immunofluorescent Staining of Macrophages

For immunofluorescent labeling, tissue sections were incubated in 10% bovine serum albumin containing 0.4% Triton X-100 for 1 h to block nonspecific binding, followed by overnight incubation at 4 °C with primary antibodies diluted in blocking solution. The primary antibodies used were anti-CD68 (1:200; Serotec, Hercules, CA, USA) and anti-Iba1 (1:100; Bioss, Woburn, MA, USA). After washing three times with PBS, sections were incubated with Alexa Fluor 488- or 594-conjugated secondary antibodies (1:500; Abcam, Cambridge, UK) in the dark for 1 h at room temperature. Slides were mounted using aqueous-based mounting medium (ScyTek, Logan, UT, USA), and fluorescence images were acquired using a confocal microscope (Leica SP2/SP8X, Wetzlar, Germany).

### 4.11. Image Analysis

Quantitative image analysis was performed using Image-Pro Lite software version 5.1 (Media Cybernetics, Rockville, MD, USA) coupled to a light microscope. CGRP-immunoreactive areas in the dorsal horn of the ipsilateral lumbar spinal cord were identified according to previously described criteria [8]. Immunoreactivity was considered positive when signal intensity exceeded background levels by at least fivefold. The proportion of CGRP-positive regions was quantified at 400× magnification. Myelinated axon density was determined by analyzing at least 50% of each sciatic nerve cross-section randomly selected from each specimen. The total number of axons was quantified using area-based algorithms at 40× magnification. Macrophage density was calculated by dividing the number of macrophages by the total nerve area, and the number of endoneurial blood vessels was counted at 400× magnification.

### 4.12. Gastrocnemius Muscle Analysis

At the end of the experimental period, both the operated (right) and contralateral (left) gastrocnemius muscles were carefully dissected and harvested. Surrounding connective tissues were gently removed, and the wet weight of each muscle was measured immediately using an analytical balance. The muscle weight ratio (operated side/contralateral side) was calculated to assess the degree of muscle atrophy.

For histological evaluation, muscle samples were fixed in 10% neutral-buffered formalin, dehydrated, and embedded in paraffin. Serial sections (5 μm thickness) were prepared and stained with hematoxylin and eosin (H&E). Muscle fiber morphology and structural changes were examined under a light microscope.

### 4.13. Quantitative Real-Time PCR (qRT-PCR)

Total RNA was extracted from regenerated nerve tissues using the RNeasy Mini Kit (QIAGEN, Hilden, Germany) according to the manufacturer’s instructions. RNA concentration and purity were determined using a NanoDrop One spectrophotometer (Thermo Scientific, Waltham, MA, USA). For selected experiments, RNA isolation was also performed using the MagNA Pure Compact RNA Isolation Kit (Roche, Boston, MA, USA) following the supplier’s protocol. Complementary DNA (cDNA) was synthesized from total RNA using the High-Capacity cDNA Reverse Transcription Kit (Applied Biosystems, Foster City, CA, USA). Reverse transcription was performed at 37 °C for 120 min according to the manufacturer’s standard protocol. Gene-specific primers corresponding to rat gene sequences were used for amplification. All oligonucleotide primers were synthesized by Mission Biotech Co., Ltd. (Taiwan). The primer sequences are listed in Table 1.

Quantitative PCR was performed using 2× Power SYBR Green PCR Master Mix (Applied Biosystems, USA) with 200 nM of forward and reverse primers. The amplification protocol consisted of an initial denaturation step at 95 °C for 10 min, followed by 40 cycles of 95 °C for 15 s and 60 °C for 1 min. Reactions were conducted in triplicate using either an Applied Biosystems 7300 Real-Time PCR System or a 7900HT Real-Time PCR System (Applied Biosystems, USA).

Gene expression levels were calculated using the comparative CT (2^−ΔΔCT^) method. GAPDH served as the endogenous internal control to normalize mRNA expression levels. The control group (“TA-A”) was used as the calibrator. Expression levels were calculated as the mean of triplicate measurements and are presented as fold changes relative to the control group.

### 4.14. Statistical Analysis

All experimental data were collected by a blinded observer and are presented as mean ± standard deviation (SD). Statistical analyses were performed using one-way analysis of variance (ANOVA) to compare differences among multiple groups. When a significant overall effect was observed, Tukey’s post hoc test was applied for multiple comparisons between groups. Prior to ANOVA, data distribution normality and homogeneity of variance were assessed. A *p*-value < 0.05 was considered statistically significant. All statistical analyses were performed using SAS 9.4 software (SAS Institute, Cary, NC, USA).

## 5. Conclusions

In summary, BVSM-crosslinked gelatin conduits provide a mechanically stable, biocompatible, and easily fabricated platform for peripheral nerve regeneration. When combined with low-frequency (2 Hz) electrical stimulation, these conduits significantly enhance axonal growth, neuronal reconnection, electrophysiological integrity, and motor–sensory functional recovery compared with unstimulated or high-frequency conditions.

Statistical analyses confirmed that 2 Hz stimulation produced significant improvements across morphometric parameters (*p* < 0.05), whereas higher frequencies (20 and 200 Hz) did not confer additional benefit and, in some cases, were less effective. The findings demonstrate a bell-shaped frequency–response relationship, indicating that low-frequency stimulation optimally balances neural activation and regenerative signaling without inducing overstimulation-related stress.

Overall, 2 Hz electrical stimulation emerges as the optimal frequency for comprehensive peripheral nerve repair, effectively promoting structural regeneration, neuroimmune coordination, neurochemical support, and functional restoration. This synergistic integration of material design and bioelectrical modulation establishes BVSM-based conduits as promising candidates for next-generation bioelectronic interfaces aimed at restoring peripheral nerve function.

## Figures and Tables

**Table 1:** Quantitative PCR primer sequences.

Gene	Forward Primer	Reverse Primer	Amplicon Size (bp)	GenBank Accession No.
NGF	GTG GAC CCC AAA CTG TTT AAG AA	AGT CTA AAT CCA GAG TGT CCG AAG A	100	NM_001277055.1
FGF	ACG GCG TCC GGG AGA A	AGG TAC CGG TTC GCA CAC A	100	NM_019305.2
PDGFA	AGG ATG CCT TGG AGA CAA ACC	TCA ATA CTT CTC TTC CTG CGA ATG	100	NM_001436393.1
TNF-α	GGC TGC CCC GAC TAC GT	AGG GCA AGG GCT CTT GAT G	102	NM_012675.3
IL-1β	GCA CCT TCT TTT CCT TCA TCT TTG	TGC AGC TGT CTA ATG GGA ACA T	100	NM_031512.2
BDNF	CAT CTG TTG GGG AGA CGA G	AAG TTG CCT TGT CCG TGG	176	NM_012513.4
TGF-β	CAC CGG AGA GCC CTG GAT A	TCC AAC CCA GGT CCT TCC TA	100	NM_021578.2
GAPDH	CTA CCC CCA ATG TAT CCG TTG T	AGC CCA GGA TGC CCT TTA GT	120	NM_017008

## Data Availability

The original contributions presented in the study are included in the article; further inquiries can be directed to the corresponding authors.

## Abbreviations

The following abbreviations are used in this manuscript:

BVSM	bisvinyl sulfonemethyl
ES	electrical stimulation
NGCs	nerve guidance conduits
FG	Fluoro-Gold
DRG	dorsal root ganglia
NCV	nerve conduction velocity
CMAP	compound muscle action potential
MAP	motor action potential
